# Evaluation of CD146 as Target for Radioimmunotherapy against Osteosarcoma

**DOI:** 10.1371/journal.pone.0165382

**Published:** 2016-10-24

**Authors:** Sara Westrøm, Tina B. Bønsdorff, Nasir Abbas, Øyvind S. Bruland, Thora J. Jonasdottir, Gunhild M. Mælandsmo, Roy H. Larsen

**Affiliations:** 1 Oncoinvent AS, Oslo, Norway; 2 Department of Tumor Biology, Institute for Cancer Research, The Norwegian Radium Hospital, Oslo University Hospital, Oslo, Norway; 3 Institute of Clinical Medicine, University of Oslo, Oslo, Norway; 4 Department of Oncology, The Norwegian Radium Hospital, Oslo University Hospital, Oslo, Norway; 5 Sciencons AS, Oslo, Norway; Duke University School of Medicine, UNITED STATES

## Abstract

**Background:**

Osteosarcoma is a rare form of cancer but with a substantial need for new active drugs. There is a particular need for targeted therapies to combat metastatic disease. One possible approach is to use an antibody drug conjugate or an antibody radionuclide conjugate to target the osteosarcoma metastases and circulating tumor cells. Herein we have evaluated a radiolabeled monoclonal antibody targeting CD146 both *in vitro* and *in vivo*.

**Methods and Results:**

A murine monoclonal anti-CD146 IgG1 isotype antibody, named OI-3, was developed along with recombinant chimeric versions with human IgG1 or human IgG3 Fc sequences. Using flow cytometry, selective binding of OI-3 to human osteosarcoma cell lines OHS, KPDX and Saos-2 was confirmed. The results confirm a higher expression level of CD146 on human osteosarcoma cells than HER2 and EGFR; antigens targeted by commercially available therapeutic antibodies. The biodistribution of ^125^I-labeled OI-3 antibody variants was compared with ^125^I-labeled chimeric anti-EGFR antibody cetuximab in nude mice with subcutaneous OHS osteosarcoma xenografts. OI-3 was able to target CD146 expressing tumors *in vivo* and showed improved tumor to tissue targeting ratios compared with cetuximab. Subsequently, the three OI-3 variants were conjugated with p-SCN-Bn-DOTA and labeled with a more therapeutically relevant radionuclide, ^177^Lu, and their biodistributions were studied in the nude mouse model. The ^177^Lu-labeled OI-3 variants were stable and had therapeutically relevant biodistribution profiles. Dosimetry estimates showed higher absorbed radiation dose to tumor than all other tissues after administration of the chimeric IgG1 OI-3 variant.

**Conclusion:**

Our results indicate that CD146 can be targeted *in vivo* by the radiolabeled OI-3 antibodies.

## Background

Osteosarcoma (OS) is the most common malignant primary tumor of bone [1] and is characterized by the presence of micrometastases in the vast majority of patients. Recurrent disease is associated with chemotherapy resistant circulating tumor cells [2–4] and metastases are particularly abundant in the lungs. The prognosis is dismal for patients with overt metastases at primary diagnosis and patients with recurrent OS have a post-relapse survival of only 20–30% [5–7]. To improve the outcome for these patient groups there is a need for new second line therapies [8]. With today’s increasing focus on personalized medicine in cancer therapy, exploiting antibodies that target cancer related antigens is one approach. In this perspective, and since OS is a relatively rare cancer [1], it may be of interest to evaluate antigens with a cross-expression on several cancers. This will improve the chances for successful clinical development of a potential product, as it is often more difficult to fund a costly development program when the patient population is limited. The tumor-associated antigens HER2 and EGFR are both known to be expressed in OS [9, 10]. Marketed immunotherapeutic antibodies targeting these antigens (trastuzumab and cetuximab) are successful for treatment of other cancers, and could be of relevance for targeted therapy of OS [11]. Trastuzumab and cetuximab have been evaluated in combination with chemotherapy in phase I studies including patients with OS [12, 13], but so far no clinical benefit has been reported.

In the present study we have assessed a recently developed murine anti-CD146 antibody and its chimeric variants [14]. CD146 is a cancer associated cell surface glycoprotein found to be expressed at elevated levels in several cancer forms including melanoma, breast cancer, prostate cancer, non-small cell lung cancer, ovarian cancer, liver cancer, mesothelioma, and OS [15–22]. CD146, also named MUC18 or MCAM, is associated with tumor progression in several of the mentioned cancers [19, 23, 24], and has been shown to act as a receptor in promotion of angiogenesis and vascular development [25]. Hence, CD146 has been suggested as a promising target for immunotherapy [26–28]. *In vivo*, treatment with an anti-CD146 antibody was found to inhibit tumor growth and metastasis in mouse models of human melanoma [29] and human OS [22]. ABX-MA1, a humanized antibody developed by Abgenix [29], entered a phase I study enrolling patients with malignant melanoma in 2002. The trial was discontinued shortly before Amgen acquired Abgenix in 2005 [30]. More recently, another anti-CD146 antibody, AA98 [31], was announced for drug candidate development against various cancers by MicroConstants China [32].

Monoclonal antibodies (mAbs) can by themselves show antitumor activity but often the effect is clinically modest and therefore antibodies conjugated to drugs, toxins or radionuclides have been used to improve antitumor activity. The use of antibody-toxin conjugates has previously been suggested as a modality in OS [33]. *In vitro* and *in vivo* targeting with radiolabeled antibodies have also been evaluated with the OS specific mAbs TP-1 and TP-3 [34]. TP-1 and TP-3 were found to bind to an alkaline phosphatase isoform with no or very limited expression on normal cells and other cancer forms [35]. It has previously been shown that the OS cell lines used in this study all express the OS specific antigen recognized by TP-3 [36].

In this study we have evaluated CD146 as a target for radioimmunotherapy of OS with the recently developed mAb OI-3. The purpose was to explore the targeting potential of our novel radioimmunoconjugate (RIC) *in vitro* and *in vivo*.

## Materials and Methods

### Antibodies

A murine monoclonal IgG1 antibody, named OI-3, was developed by standard hybridoma technologies [14]. Chimeric variants of the murine antibody with human IgG1 (CHOI-3.1) or IgG3 (CHOI-3.3) Fc portion were developed by recombinant cloning of the heavy and light variable chains of OI-3 with human IgG/κ light chain and IgG1 or IgG3 heavy chain [14]. Chimeric anti-EGFR IgG1 mAb cetuximab (Erbitux, Merck, Darmstadt, Germany) and humanized anti-HER2 IgG1 mAb trastuzumab (Herceptin, Roche, Basel, Switzerland) were used as antigen positive controls for co-expressed antigens in flow cytometry analyses. Chimeric anti-CD20 IgG1 mAb rituximab (MabThera, Roche) and murine anti-CD37 IgG1 mAb HH1 (also named tetulomab, Diatec Monoclonals, Oslo, Norway) [37, 38] were used as antigen negative controls. HH1 and cetuximab were also used in biodistribution studies.

### Cell lines

Human OS cell lines OHS [39], KPDX [35, 40] and Saos-2 (purchased from ATCC) were used in the study. The cells were cultured in monolayer in RPMI 1640 (Life Technologies Invitrogen, Thermo Scientific, Waltham, MA, USA) supplemented with 10% Fetal Calf Serum and 1% Penicillin/Streptomycin at 37°C in a humid atmosphere with 5% CO_2_. Cultures were propagated and harvested at 80–90% confluence by detachment with TrypLe Express (Life Technologies). All cell lines were authenticated via STR profiling performed by the ATCC Cell Authentication Testing Service using Promega’s PowerPlex® 18D System. The resulting STR profiles either matched the ATCC reference database profile (Saos-2) or were unique (OHS and KPDX). Furthermore, the expression status of the OS associated antigen recognized by TP-3 was verified for all three cell lines.

### Measurement of CD146 expression by flow cytometry

The OS cell lines were harvested and cell numbers were determined by utilizing the Countess Cell Counter (Invitrogen) followed by dilution in flow buffer (Dulbecco’s PBS with 0.5% BSA and 0.1% NaN_3_). The cell suspension was distributed in a 96 well plate with 2 × 10^5^ cells per sample well. Primary antibodies were added in a concentration of 10 μg/ml and cells were incubated at 4°C for 30 min before three washes in 200 μl flow buffer. Secondary antibody, anti-mouse IgG F(ab’)2 fragment-FITC (Sigma-Aldrich, St Louis, MO, USA) or anti-human IgG-FITC (US Biologicals, Salem, MA, USA), was added and incubated for 30 min and washed as in the previous step. All wash steps were performed by centrifugation at 1,200 rpm for 5 min.

Washed cell pellets were dissolved in flow buffer and analyzed in a FACS Calibur (BD Bioscience, Franklin Lakes, NJ, USA) or Guava EasyCyte HT flow cytometer (Merck Millipore, Darmstadt, Germany). The flow analysis was replicated and reproduced at least three times for all cell lines. The data were analyzed with Kaluza Analysis 1.3 software (Beckman Coulter, Brea, CA, USA).

### Radiolabeling of antibodies with ^125^I and ^177^Lu

Radioiodination with ^125^I (Hartmann Analytic, Braunschweig, Germany) was carried out using pre-coated iodogen tubes (Pierce, Rockford IL, USA) according to previously published procedures [41]. After iodination, the RIC was purified by size exclusion chromatography on a Sephadex G-25 PD10 column (GE Healthcare Biosciences AB, Uppsala, Sweden). The radiolabeling yields were typically 65–90% and specific activity was between 20–50 MBq/mg.

For radiolabeling with ^177^Lu, the antibodies were first conjugated to a chelator, p-SCN-Bn-DOTA (Macrocyclics, Dallas, TX, USA). The conjugation was performed as described by Repetto-Llamazares *et al*. [41], but with minor modifications. Briefly, p-SCN-Bn-DOTA was dissolved in 5 mM HCl and added to the antibody in a molar ratio of approximately 5:1. The reaction was pH adjusted to approximately 8.5 using 0.1 M carbonate buffer. After 2 h incubation at 20°C with gentle shaking, unconjugated chelator was separated from DOTA-conjugated antibody and the buffer exchanged to 0.9% NaCl using a centrifuge filtering cartridge (Vivaspin 15R, 50 kDa MWCO, Sartorius Stedim Biotech, Göttingen, Germany). To prepare the RIC, ^177^Lu in 10 mM HCl (ITG, Garching, Germany) was mixed with DOTA-conjugated antibody in 0.5 M ammonium acetate (to adjust the pH to 5.5–6.0) and reacted for 15–45 min at 37°C. The radiochemical purity of the resulting product was evaluated with chromatography strips (Biodex, Shirley, NY, USA), and if the purity was below 95% the conjugate was purified using a PD10 gel filtration column. The radiochemical purity was above 96% for all ^177^Lu conjugates used in the study.

### Immunoreactive fraction and affinity measurements

The immunoreactive fractions of the radiolabeled antibodies were determined in one-point binding assays *in vitro*. A single cell suspension of 25–50 × 10^6^ cells/ml of Dulbecco’s PBS supplemented with 0.5% BSA was prepared. Duplicate samples of 0.2 ml cell solution were incubated at room temperature for about 60 min with gentle shaking together with radiolabeled mAb (1–4 ng). Nonspecific binding was estimated from samples preincubated (15–30 min) with 20 μg unlabeled mAb prior to the addition of radiolabeled mAb. The total radioactivity in each sample was measured in a gamma counter (Cobra II auto-gamma detector, Packard Instrument Company, Meriden, CT, USA). After centrifuging and washing the cells three times in Dulbecco’s PBS with 0.5% BSA, measurements on the cell pellets determined the cell-bound radioactivity. The fraction of bound mAb was calculated by dividing the amount of cell-bound activity on the total added radioactivity, and the immunoreactivity was the fraction of bound mAb minus the fraction of unspecific bound mAb. The immunoreactive fraction was above 50% for all conjugates used in the experiments.

Saturation binding experiments were set up to determine the equilibrium dissociation constant, K_D_, and the number of specific binding sites, B_max_, for the RICs on OHS cells. Increasing amounts of ^125^I-OI-3 or ^177^Lu-labeled versions of CHOI-3.1, CHOI-3.3 or cetuximab, ranging from 10 ng to 20 μg per ml, were added to 0.1 ml samples of 10 × 10^6^ cells/ml of Dulbecco’s PBS with 0.5% BSA, four samples of each concentration. Incubation with gentle agitation was allowed to proceed for 60–90 min at room temperature, or 4°C for ^125^I-OI-3. Unspecific binding was determined by letting half of the samples preincubate for 30–60 min in the presence of approximately 100 μg/ml unlabeled mAb, before the RIC was added. Measurements of the total and cell-bound activity were carried out as previously described for the immunoreactivity assay. Values of K_D_ and B_max_ were determined from globally fitting the experimental binding data to the sum of a hyperbolic curve (specific binding) and a straight line (nonspecific binding):

$$B=\frac{B_{max}[T]}{K_{D}+[T]}+NS\times [T]$$

where B is the number of antigen sites per cell, [T] is the total concentration of RIC corrected for the corresponding immunoreactive fraction and NS is the slope of the nonspecific binding. The fitting was performed with GraphPad Prism version 6.00 for Windows (GraphPad Software, La Jolla, CA, USA).

### Animals

Institutionally bred, 4–8 weeks old, female Athymic nude *Foxn*^*nu*^ mice with body weights in the range of 18–25 g at the start of the experiment were used. Animals were maintained under pathogen-free conditions with food and water supplied *ad libitum*. OHS tumor fragments (approximately 2×2×2 mm in size) from *in vivo* passages were implanted subcutaneously on the rear flanks of the mice under sevoflurane anesthesia. Mice were monitored up to twice weekly for changes in tumor size, bodyweight, behavior, posture and appearance. Humane endpoints were a tumor volume of 2000 mm^3^, evident skin necrosis and/or ulceration over the tumor, weight loss above 15% or other signs of distress and/or discomfort. Animals that reach one of these endpoints were euthanized by cervical dislocation. No animals became ill or died prior to the experimental endpoint, and only the tumor related endpoint criteria were used.

All procedures and experiments involving animals were approved by the National Animal Research Authority (permit ID 5639 and 5734) and were performed in accordance with the European Convention for the Protection of Vertebrates Used for Experimental and other Scientific Purposes [42].

### Biodistribution of radiolabeled antibodies

The RICs were administered by tail vein injection of 100 μl solution to mice bearing OHS xenografts with largest tumor diameter between 4 and 17 mm. At different time points post injection (from 6 h up to 14 days) blood was collected by cardiac puncture while the mice were under sevoflurane anesthesia. Immediately after blood sampling, the animals were euthanized by cervical dislocation, before tumor and different tissues (lungs, heart, liver, spleen, kidney, stomach, small intestine, large intestine, femur, muscle, brain and skull) were removed at the autopsy. Each tissue sample was weighed, and the radioactivity measured in a gamma counter (Cobra II auto-gamma detector). Samples of the injectates were used as a reference in the measurement procedures. The decay corrected percentages of the injected dose per gram tissue (% ID/g) were calculated for all time points, and plotted with GraphPad Prism. A group size of three to six animals was used per time point. Tumors with weight less than 50 mg were excluded from the analyses.

### Dosimetry estimates

The biodistribution data for tumor, blood and a panel of normal tissues were used for estimation of absorbed radiation dose from ^177^Lu-CHOI-3.1, assuming a standard dosing of 1 MBq per mouse (*i*.*e*., 40 MBq/kg for a 25 g mouse). Absorbed radiation dose from time of injection until no radioactivity remains in the body was calculated as described by Yuan [43]. The area under the curve from time point 0 to 14 days was calculated by the trapezoidal rule in GraphPad Prism under the assumption of activity at time t = 0 was 100% of total injected activity in blood, and 0 in all other tissues. Extrapolation beyond the last time point (14 days) was done by assuming that clearance of radioactivity from this time point was only due to radioactive decay. The absorbed radiation dose from ^177^Lu-CHOI-3.1 was obtained by multiplying the area under the specific activity versus time curve with 0.1473 MeV, which corresponds to the mean energy of beta particles, Auger- and conversion electrons from the decay of ^177^Lu [44].

## Results

### CD146 expression in human OS cell lines

**Fig 1:**
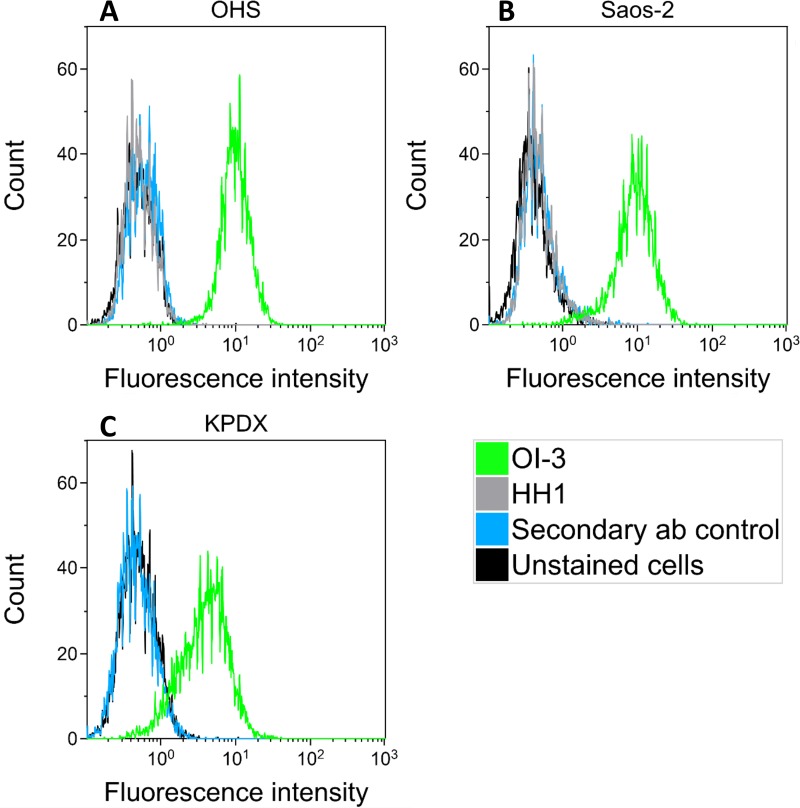
CD146 expression in human osteosarcoma cell lines. A representative flow cytometry histogram that compare the binding of murine anti-CD146 OI-3 antibody to three different human osteosarcoma cell lines: OHS (A), Saos-2 (B) and KPDX (C). Control samples are unstained cells and cells stained only with the FITC-conjugated secondary antibody (ab). In addition, the binding of murine anti-CD37 antibody HH1 was examined for OHS (A) and Saos-2 (B) cells. The samples were analyzed on a BD FacsCalibur.

Surface expression of CD146 on the OHS, Saos-2 and KPDX cells was confirmed with flow cytometry analyses and a strong, specific binding of the murine OI-3 mAb was shown (Fig 1). The well-defined narrow histogram shape of OI-3 binding to OHS and Saos-2, with good separation to the control samples, indicates a high and relatively uniform expression of CD146 on these cell lines (Fig 1A and 1B). The histogram peak is less intense and the shape is broader for OI-3 binding to KPDX (Fig 1C), indicating a slightly lower and more heterogeneous expression of CD146 in this cell population. A small subset of KPDX cells shows no expression of CD146, as the histogram overlap to some extent with the secondary antibody control and unstained cell samples. No expression of CD37 was detected on OHS and Saos-2 as HH1 showed identical staining to that of the secondary antibody control and unstained cell samples.

**Fig 2:**
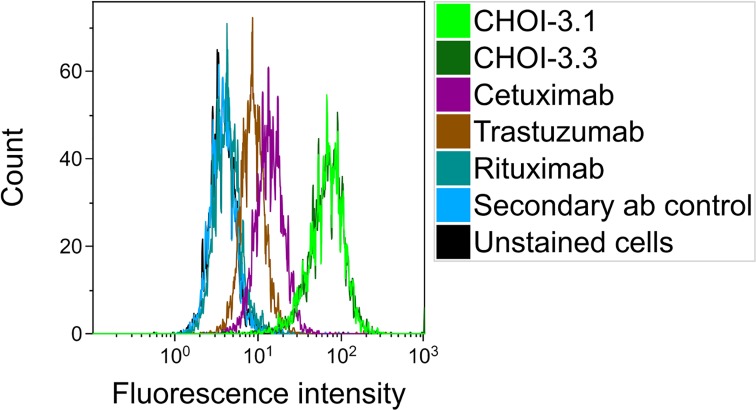
Comparison of the binding ability of chimeric antibodies targeting different antigens on OHS cells. A representative flow cytometry histogram which shows binding of the chimeric versions of OI-3 to OHS cells, compared with samples incubated with cetuximab, trastuzumab and rituximab. Control samples are unstained cells and cells stained only with the FITC-conjugated secondary antibody (ab). The samples were run on a Guava EasyCyte HT. Take note that the samples with chimeric antibodies were run on a different platform, with different settings and secondary antibody, and the fluorescence intensity levels are therefore not directly comparable to runs with murine OI-3 in Fig 1.

The chimeric versions of OI-3; CHOI-3.1 and CHOI-3.3, were found to bind to the OHS cell line in a similar way as the murine OI-3 mAb (Fig 2), giving additional confirmation of high CD146 expression on OHS. Samples of OHS stained with the commercial antibodies cetuximab, trastuzumab and rituximab were also included in the analysis and showed that EGFR and HER2 are clearly present on the plasma membrane of OHS cells, whereas CD20 is not. Interestingly, the results revealed that CD146 had a higher expression than both EGFR and HER2 on the OHS cell line. OHS was therefore chosen for further studies, both because of its strong CD146 expression and the possibility to compare the chimeric OI-3 antibody to commercially available antibodies against well-known targets.

### Affinity measurements

The equilibrium dissociation constant, K_D_, and the mean number of binding sites, B_max_, were determined for the OI-3 variants and cetuximab on the OHS cell line. The K_D_ were in the same order of magnitude for the OI-3 variants, with values of 1.8 ± 1.2, 2.6 ± 0.3 and 8.2 ± 5.1 nM for OI-3, CHOI-3.1 and CHOI-3.3 respectively. Cetuximab was determined to have a K_D_ of 0.14 ± 0.1 nM on OHS cells, indicating a slightly better affinity for the EGFR antigen than the OI-3 variants had for the CD146 antigen. The OHS cells showed a relatively high expression of CD146 antigens, an average of (1.65 ± 0.96) × 10^5^ sites per cell were found. In accordance with the results from the flow cytometry analyses, the average expression of EGFR on the OHS cell line were found to be lower than for CD146, with only 9921 ± 2855 sites per cell. Altogether the results from the affinity experiments show a notable difference in affinity and number of antigen sites for the OI-3 variants in comparison with cetuximab.

### Biodistribution of ^125^I-labeled monoclonal antibodies in OHS xenografts

**Fig 3:**
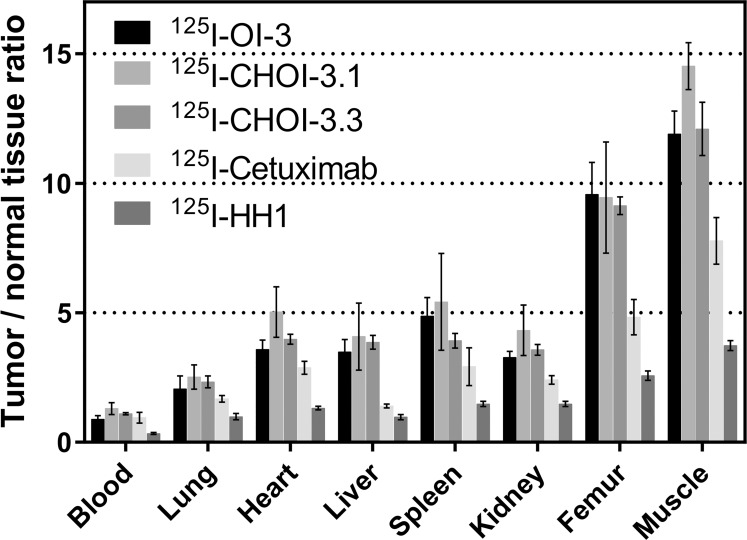
Biodistribution of ^125^I-labeled antibodies in mice bearing OHS xenografts. Tumor to normal tissue ratios 24 hours after injection of ^125^I-labeled antibodies in female nude mice bearing OHS tumor xenografts. Four to five animals were used for each radioimmunoconjugate, which gave from four to seven tumors per group. Error bars correspond to standard error of the mean.

The biodistribution of ^125^I-labeled mAbs in nude mice bearing OHS xenografts was evaluated 24 hours after injection to determine the ability of OI-3 to target CD146 expressing tumors *in vivo*. The murine OI-3 mAb was compared with antigen negative control HH1, and CHOI-3.1 and CHOI-3.3 were compared with cetuximab. Fig 3 presents the data as tumor to normal tissue ratios for the five different ^125^I-labeled RICs. The tumor to blood ratios were similar for all five RICs, but tumor to tissue ratios were higher for the three OI-3 variants compared to cetuximab. As expected, HH1 had the lowest tumor to tissue ratios since it does not selectively bind to any antigen in the tumor.

### Biodistribution of ^177^Lu-labeled OI-3-variants in OHS xenografts

**Fig 4:**
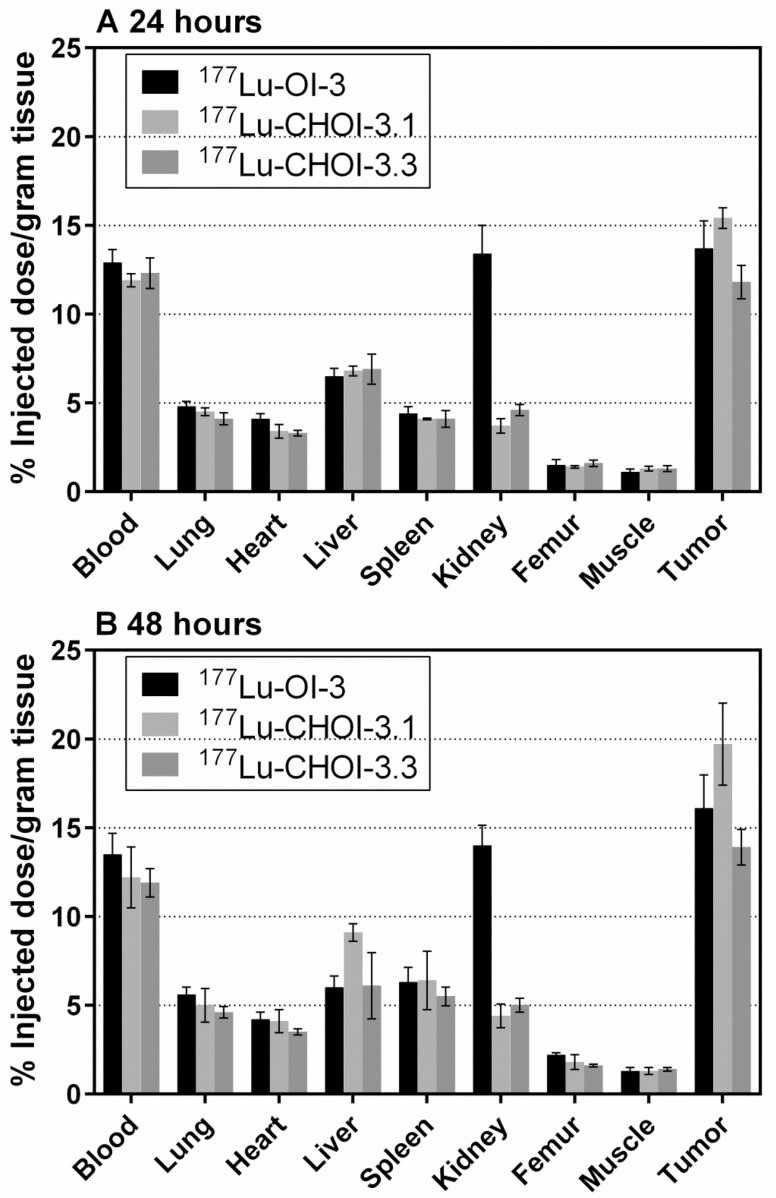
Biodistribution of ^177^Lu-OI-3 variants in mice bearing OHS xenografts. Comparison of biodistribution of ^177^Lu-labeled murine OI-3, chimeric IgG1 OI-3 (CHOI-3.1) and chimeric IgG3 OI-3 (CHOI-3.3) in nude mice with OHS osteosarcoma xenografts. The data are presented as percentage of injected dose per gram tissue at 24 (A) and 48 hours (B) after injection, with error bars corresponding to the standard error of the mean. Three to six mice were used in each group, giving four to eight tumors per time point.

Fig 4 shows the biodistribution of ^177^Lu-labeled versions of OI-3 in nude mice bearing OHS xenografts at 24 and 48 hours after injection. Comparing the three conjugates, the data are similar but it tended to be slightly better tumor uptake for CHOI-3.1. At both time points, the percentage of injected dose per gram tissue was higher for tumor than any of the normal tissues, giving favorable tumor to non-tumor ratios for all ^177^Lu-labeled OI-3 variants. The only exception was in the case of the murine conjugate, where relatively high activity was measured in the kidneys. Several mice had comparable uptake in kidneys and tumors, which is evident in a low tumor to kidney ratio of slightly above one. Note that the uptake in femur was low, indicating no free ^177^Lu, *i*.*e*. all RICs had a high stability *in vivo*.

**Fig 5:**
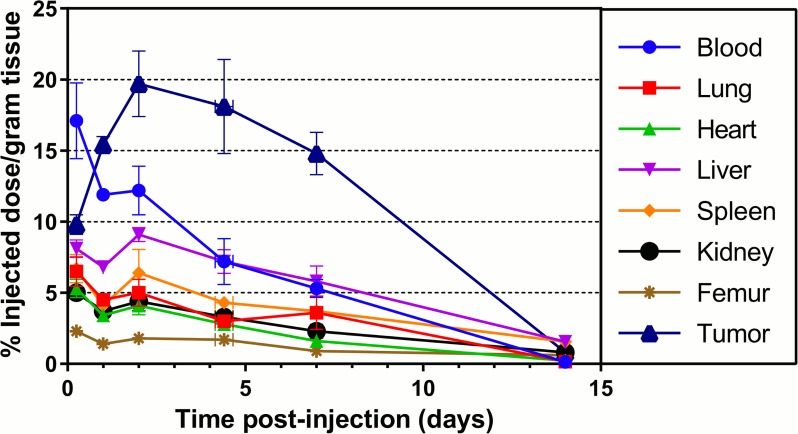
Biodistribution of ^177^Lu-CHOI-3.1 in mice bearing OHS xenografts. Biodistribution of ^177^Lu-labeled chimeric OI-3 IgG1 isotype antibody (CHOI-3.1) in tissues of interest in OHS xenograft-carrying nude mice. At each time point from three to six animals were used, with number of tumors ranging from five to twelve per group. Straight lines have been drawn to connect the data points. The error bars correspond to the standard error of the mean.

As most commercially available mAbs are of the IgG1 isotype, the biodistribution of ^177^Lu-labeled CHOI-3.1 was studied at additional time points. The distribution of radioactivity in various tissues as a function of time after administration of the ^177^Lu-labeled CHOI-3.1 is shown Fig 5. It is seen that the RIC is rapidly taken up in most tissues. However, at all time points the percentage of injected dose per gram tissue was higher for tumor than any other normal organ. The radioactivity in the tumors increased up to 2 days after injection, with a maximum uptake between 2 and 4 days, whereas the uptake in normal tissues decreased or remained essentially unchanged during the same course of time.

### Dosimetry

**Fig 6:**
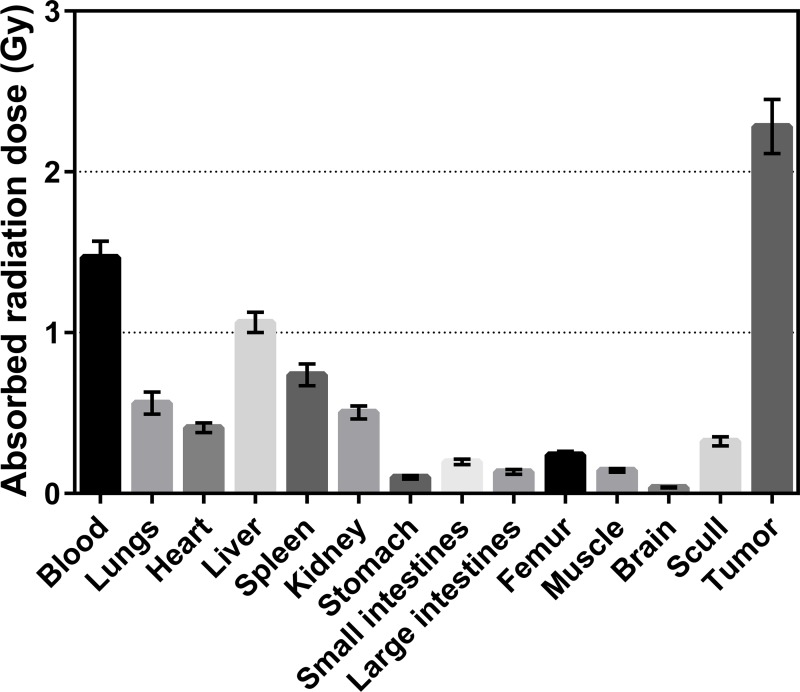
Absorbed radiation doses to normal tissues and tumors. Estimated absorbed radiation dose (Gy) to normal tissues and tumors for nude mice with OHS osteosarcoma xenografts after intravenous injection of ^177^Lu-labeled chimeric OI-3 IgG1 isotype antibody (CHOI-3.1). The data were normalized to an injected activity of 1 MBq per mouse. Error bars correspond to standard deviation.

Fig 6 presents the absorbed radiation dose assessment for ^177^Lu in mice based on the biodistribution data of ^177^Lu-labeled CHOI-3.1 when the injected activity was normalized to 1 MBq/mouse. As illustrated, the absorbed radiation dose to tumor is considerably higher than to any other tissue or blood. The doses were below 1.1 Gy to all normal tissues, whereas for tumor it was 2.3 Gy. About 90% or more of the radioactive atoms had cleared or decayed from the blood and tissues after 14 days, therefore the uncertainty introduced by not including later time points in the biodistribution measurements are considered to be minor.

## Discussion

The current study shows that the OI-3 mAbs target an epitope on the CD146 antigen which is readily accessible both *in vitro* and *in vivo*. Overexpression of CD146 was confirmed in the three human OS cell lines tested *in vitro*, a result which is consistent with reports from the literature of CD146 expression in other OS cell lines [22, 45]. The biodistribution studies also demonstrate that the three OI-3 variants bind with high specificity to OHS xenografts in nude mice, confirming that CD146 is well expressed *in vivo* by these tumors. OHS is well established as a tumor line in mice [39, 46, 47]. Therefore, the OHS xenograft model was chosen to evaluate the stability and targeting potential of the radiolabeled OI-3 variants. *In vitro* affinity studies of the OI-3 mAbs support that OHS is a suitable cell line with a high CD146 expression level, and relevant range of affinity for all radiolabeled OI-3 variants. The tendency for variations in the measured K_D_ of the OI-3 variants can reflect differences caused by quaternary structures of for instance IgG1 and IgG3 sequences. Comparative studies on OHS show that the estimated number of CD146 antigens is approximately 15 times higher than that of EGFR antigens, whereas the affinity of cetuximab binding is higher. Although the data presented here and by others support that human OS cell lines have therapeutic relevant CD146 expression, it is important to emphasize that antigen expression on human cell lines not necessarily reflects the situation in patients. However, CD146 expression has been reported as a marker for vascularization in tissue sections from biopsies of OS patients [48] and as a marker for tumor-propagating capacity in primary human OS cells [49]. An anti-CD146 antibody has been examined as an immunotherapeutic agent for treatment of melanoma [26, 27], and has been reported to inhibit spontaneous pulmonary metastasis of OS cells in mice [22]. Unlike existing literature describing the immunotherapeutic potential of different anti-CD146 antibodies, we have focused on utilizing our recently developed anti-CD146 mAb as a RIC for targeting of OS.

The presented biodistribution data indicate that favorable tumor to tissue ratios can be achieved with radiolabeled versions of the OI-3 mAbs, indicative of CD146 being a suitable target for radioimmunotherapy. In the OHS xenograft model, 24 hours after intravenous administration of ^125^I-labeled mAbs TP-1 and TP-3 [34, 50], tumor to normal tissue ratios were comparable to the ratios found for the OI-3 mAbs labeled with ^125^I in this study. The results obtained when the OI-3 mAbs were labeled with the more therapeutically relevant radionuclide ^177^Lu, further demonstrated a promising biodistribution profile. The uptake and retention of ^177^Lu-CHOI-3.1 over time in the tumors vs. blood and normal tissues indicated substantially higher radiation to tumor vs. normal tissues in the nude mice model.

CD146 is known to be expressed at some level in endothelial cells of both normal and cancer cells in human tissue sections [18]. The murine anti-human CD146 OI-3 antibody do not have affinity for the murine CD146 antigen (non-published data), and further analysis of OI-3 binding in clinical relevant models of human normal tissues is required to indicate if the promising biodistribution profile in animal models also can be expected in patients. CD146 has been shown to be expressed by both epithelioid and sarcomatous types of mesothelioma tumor cells in human tissue sections but not by normal mesothelial cells [17]. CD146 expression has been reported in a relatively limited spectrum of normal human adult tissues: Endothelium, smooth muscle, Schwann cells, ganglion cells, myofibroblasts, cerebellar cortex, breast, hair follicles, and dendritic cells [51]. Cross-reactivity with normal cells and tissues in humans can reduce antitumor activity and cause normal tissue toxicity of radioimmunotherapy. As with the previously approved RICs Zevalin and Bexxar, pre-treatment of patients with non-labeled antibodies to block binding to normal cells may be required.

Maximum uptake in tumors occurred between 2 and 4 days after administration of ^177^Lu-CHOI-3.1. This kinetics are suitable for a radioisotope with 6.7 days half-life like ^177^Lu because it decays and thereby delivers the radiation in the same period of time as the uptake of the RIC in tumor is at its highest. Conjugating the CHOI-3.1 mAb to a radioisotope with shorter half-life, e.g. the beta emitter ^90^Y with a half-life of 2.7 days, would in this case result in less favorable tumor to normal tissue ratios since ^90^Y would have decayed to approximately 50% of the initial activity before the RIC would reach maximum uptake in the OHS model, causing higher radiation doses to blood or other normal tissues instead of tumor.

The RICs were found to have relevant stabilities *in vivo*. Free ^177^Lu accumulates predominantly in bone. Repetto-Llamazares *et al*. showed that uptake of free ^177^Lu in the bones of nude mice without tumors was 10 and 13% of injected dose per gram tissue after 24 and 48 hours respectively [52], as opposed to a maximum of 2% measured for the OI-3 conjugates.

Cetuximab has been proposed for targeted therapy of OS because of expression of the EGFR antigen. Two patients with OS were enrolled in a phase I study of cetuximab and irinotecan in children with refractory solid tumors [13], although no clinical effect has been reported yet. In OS cells *in vitro* cetuximab enhanced the cytolytic activity of natural killer cells [53]. The present study support that CD146 has significant expression in the OHS cell line and xenograft making CD146 a potential target for both immunotherapy and radioimmunotherapy. Compared with ^125^I-labeled cetuximab, radiolabeled OI-3, in murine as well as chimeric versions, showed slightly improved tumor to normal tissue ratios in nude mice bearing OHS xenografts. This is an interesting observation, considering both that cetuximab is approved for clinical use and the recent interest in cetuximab as a novel treatment of OS.

In conclusion, radiolabeled versions of the OI-3 anti-CD146 mAb seem to satisfy several of the requirements for *in vivo* targeting of OS tumors. The tumor uptake in nude mice was promising, together with a low to moderate uptake in normal tissues and a high *in vivo* stability. The results warrant further evaluation of ^177^Lu-labeled OI-3 as a potential candidate for radioimmunotherapy against metastatic OS and other CD146 expressing cancers.
